# Sentinel Lymph Node Biopsy Pathology and 2-Year Postsurgical Recurrence of Breast Cancer in Kenyan Women

**DOI:** 10.1200/JGO.17.00111

**Published:** 2017-12-07

**Authors:** Nathan R. Brand, Ronald Wasike, Khalid Makhdomi, Rajendra Chauhan, Zahir Moloo, Samuel M. Gakinya, Alfred I. Neugut, Jo Anne Zujewski, Shahin Sayed

**Affiliations:** **Nathan R. Brand** and **Alfred I. Neugut**, Columbia University, New York, NY; **Ronald Wasike**, **Khalid Makhdomi**, **Rajendra Chauhan**, **Zahir Moloo**, **Samuel M. Gakinya**, and **Shahin Sayed**, Aga Khan University Hospital, Nairobi, Kenya; and **Jo Anne Zujewski**, Leidos Biomedical Research, Frederick, MD.

## Abstract

**Purpose:**

The goal of this study was to describe the pathologic findings and early follow-up experience of patients who underwent a sentinel lymph node biopsy (SLNB) at Aga Khan University Hospital (AKUH) between 2008 and 2017.

**Patients and Methods:**

We performed a retrospective analysis of women with breast cancer who underwent an SLNB at AKUH between 2008 and 2017. The SLNB was performed on patients with stage I and stage II breast cancer, and identification of the sentinel lymph node was made by radioactive tracer, blue dye, or both, per availability and surgeon preference. Demographic, surgical, and pathologic data, including immunohistochemistry of the surgical sample for estrogen receptor, progesterone receptor, and human epidermal growth factor receptor 2, were abstracted from the patient records. Follow-up data were available for a subset of patients.

**Results:**

Between 2008 and 2017, six surgeons performed SLNBs on 138 women, 129 of whom had complete records and were included in the study. Thirty-one of 129 (24%) had a positive SLNB, including 10 of 73 (14%) with stage I and 21 of 56 (38%) with stage II disease. Seventy-eight patients (60%) received systemic adjuvant chemotherapy and 79 (62%) received radiation therapy, and of the 102 patients who were estrogen receptor positive, 86 (85%) received endocrine therapy. Seventy-nine patients were observed for > 2 years, and, of these, four (5.1%) had a regional recurrence.

**Conclusion:**

The SLNB positivity rates were similar to those of high-income country (HIC) cohorts. However, preliminary data suggest that recurrence rates are elevated at AKUH as compared with those of HIC cohorts, perhaps because of a lower use of radiotherapy and chemotherapy at AKUH compared with HIC cohorts or because of differences in the characteristics of the primary tumor in patients at AKUH as compared with those in HICs.

## INTRODUCTION

Breast cancer is the most common malignancy among women worldwide, causing 522,000 deaths in 2012.^1^ Although the per capita incidence of this disease is greater in more developed countries, patient fatality rates are significantly higher in low- and middle-income countries.^1^ This has led to a significant global effort to promote early diagnosis and treatment of breast cancer in low-resource settings.^2,3^ Currently, most patients with breast cancer in low- and middle-income countries are diagnosed at an advanced stage; however, the promotion of early diagnosis and treatment is expected to lead to an increase in stage I and II diagnoses,^4^ which are associated with significantly better outcomes than are stage III or IV disease.

In Kenya, breast cancer is the most common malignant neoplasm in women, with an age-standardized incidence rate of 51.7 per 100,000 women.^5^ Although no long-term follow-up data on 5-year survival are available from Kenya, in Uganda, the reported cumulative 5-year survival is 51.8%.^6^ At Aga Khan University Hospital (AKUH), a 300-bed teaching hospital in Nairobi, Kenya, patients with stage I and II breast cancer are offered a sentinel lymph node biopsy (SLNB). The importance of this procedure in the management of early-stage breast cancer continues to be affirmed in guidelines from high-income countries (HICs),^7^ and knowledge of its importance is growing in low-resource settings as well, as demonstrated by the recent International Atomic Energy Agency’s meeting on setting up SLNB programs in developing countries.^8^ However, despite this support, many barriers to creating SLNB programs in low-resource settings, including lack of specialist training, limited resources, and equipment shortages, have been identified.^9^ In addition, limited data have been published on successful SLNB programs in Africa, and no data have been published from East Africa. In this retrospective study, we describe the pathologic findings of patients who underwent an SLNB at AKUH between 2008 and 2017 together with their experience with breast cancer recurrence in the subsequent 2 years.

## PATIENTS AND METHODS

### Hospital Site

AKUH is a not-for-profit teaching hospital in Nairobi with 300 beds and an average of 170 new breast cancer diagnoses per year. The hospital has a dedicated breast clinic and is able to offer multidisciplinary care that includes nuclear medicine, surgery, chemotherapy, radiotherapy, and pathology services, which are all coordinated through weekly breast tumor boards.

### Ethical Approval

The AKUH’s institutional review board approved this retrospective study, and a de-identified data set was created by an AKUH research nurse for the data analysis. Columbia University provided an institutional review board exemption because their personnel worked with the de-identified data only.

### Data Abstraction

Cross-referencing patient records from the surgical, nuclear medicine, and pathology departments identified patients who underwent an SLNB. AKUH study personnel abstracted data into an Excel spreadsheet (Microsoft, Seattle, WA) on all women who underwent an SLNB between 2008 and 2017 for clinical stage I or II malignant breast cancer.

### Surgeons

Six general surgeons at AKUH performed SLNBs during the study period. Of the six, two surgeons completed > 95% of the caseload. These two surgeons were both trained outside of Kenya on SLNB technique: one surgeon received training through a breast fellowship in Canada, and the other completed a surgical oncology fellowship in India. Another surgeon, who completed a breast fellowship in South Africa in 2016 and only recently returned to AKUH, contributed three cases, and three other AKUH surgeons each contributed a single SLNB case to the study.

### SLNB Procedure

The six AKUH surgeons performed the SLNB procedure on stage I and stage II patients with no nodal or metastatic disease. Nodal status was determined by physical examination, and metastatic work-up included chest and abdominal computed tomography and bone scan on all patients. The sentinel lymph node (SLN) was identified using lymphoscintigraphy with ^99m^Tc-nanocolloid and gamma probe or lymphatic dye mapping with methylene blue, or both, depending on surgeon preference and consumable availability. Identified SLNs were removed from the patient and were analyzed by a pathologist using touch prep, scrape prep, fresh-frozen sectioning, or a combination of approaches, depending on pathologist preference.

### Pathology

Postoperatively, all samples were delivered to the pathology laboratory for permanent fixation and processing. Hematoxylin and eosin–stained sections of the SLNs and the primary tumor were examined for tumor type, and histopathologic features of prognostic significance such as tumor grade and lymphovascular invasion were noted.

### Immunohistochemistry

Details of the immunohistochemistry (IHC) procedures at AKUH have been described previously.^10^ In brief, tumor sections were stained for estrogen receptor (ER; FLEX RTU monoclonal rabbit anti-human ER α, clone EP1), progesterone receptor (PR; FLEX RTU monoclonal rabbit anti-human PgR 636 antibody clone), and human epidermal growth factor receptor 2 (HER2)/neu (polyclonal rabbit anti-human c-erbB-2 oncoprotein diluted 1:200 with the EnVisio FLEX antibody diluent) on the Dako Autostainer Link 48 platforms, using EnVisio FLEX Kit detection kits (Dako, Santa Clara, CA). Interpretation for ER/PR used the Allred scoring system,^11^ and HER2 positivity was scored using the ASCO/College of American Pathologists scoring system,^12^ with equivocal samples receiving fluorescent in situ hybridization to assess gene amplification.

### Data Analysis

Data were exported to and analyzed in Stata SE 12 (StataCorp, College Station, TX). Categorical variables were analyzed using the χ^2^ test, and continuous variables were assessed using the Wilcoxon rank sum test or the *t* test, depending on the normality of the distribution of the variable.

## RESULTS

**Fig. 1:**
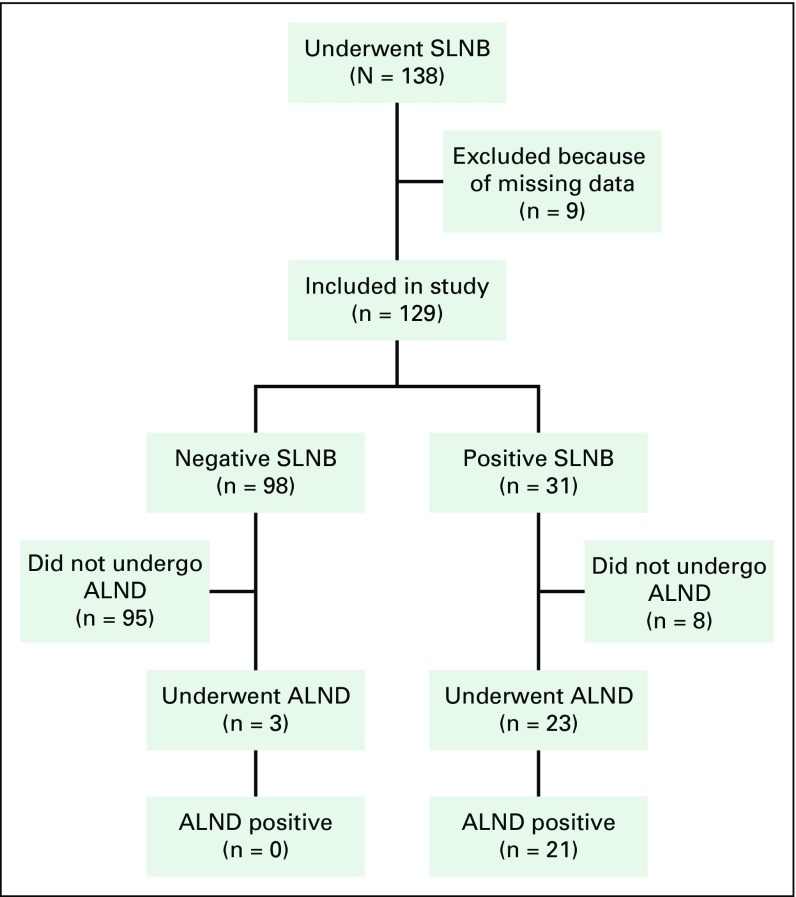
Patients who underwent sentinel lymph node biopsy (SLNB) and axillary lymph node dissection (ALND). Patients who did not undergo an ALND after a positive SLNB had two or fewer positive sentinel lymph nodes and were prescribed whole-breast radiotherapy and breast conservation therapy.

Between July 1, 2008, and April 30, 2017, 138 women underwent an SLNB by one of the six study surgeons. Nine of these patients, however, had undergone an SLNB before AKUH’s transition to an electronic medical record system and therefore were excluded from our study due to significant missing data. This left a final sample of 129 patients, including 73 with stage I and 56 with stage II disease. At least one SLN was found in 100% of these patients. A positive SLNB was found in 31 (24%) of the patients, including 10 of 73 (14%) with stage I and 21 of 56 (38%) with stage II disease. Of the 31 patients with a positive SLNB, 23 proceeded to axillary lymph node dissection (ALND) and eight were treated with an SLNB alone after a change in ASCO’s practice guidelines.^13^ Of the 23 patients with a positive SLNB who underwent an ALND, 21 (91%) were found to have one or more other positive nodes. Tables 1 and 2 list the demographic, surgical, and pathologic characteristics associated with SLN positivity and demonstrate the association between younger age, stage II disease, lymphovascular invasion, and histologic diagnosis and SLN positivity. Of the 129 patients, 78 (60%) received systemic adjuvant chemotherapy, 79 (62%) received radiation therapy, and 86 (85%) of the 102 patients with ER positivity received endocrine therapy. During the study period, three patients with a negative SLNB underwent an ALND for unknown reasons. None of these patients had a positive node on ALND (Fig 1).

**Table 1:**
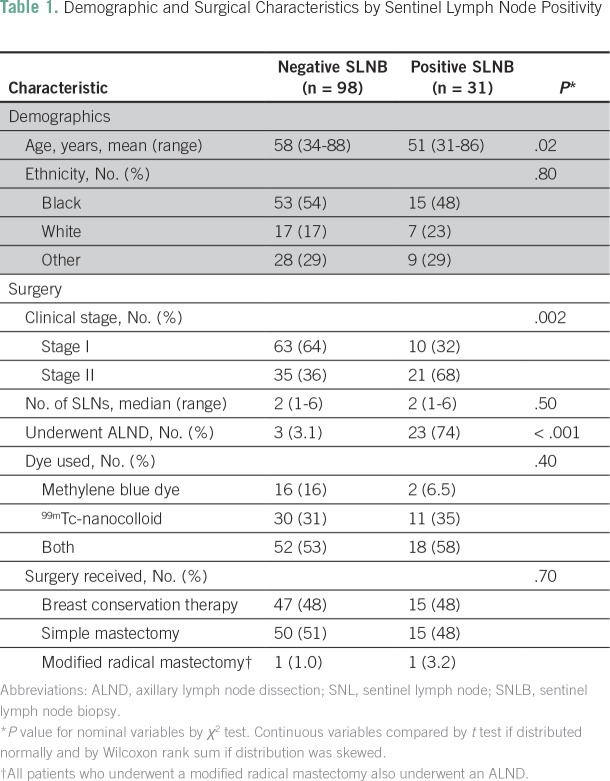
Demographic and Surgical Characteristics by Sentinel Lymph Node Positivity

**Table 2:**
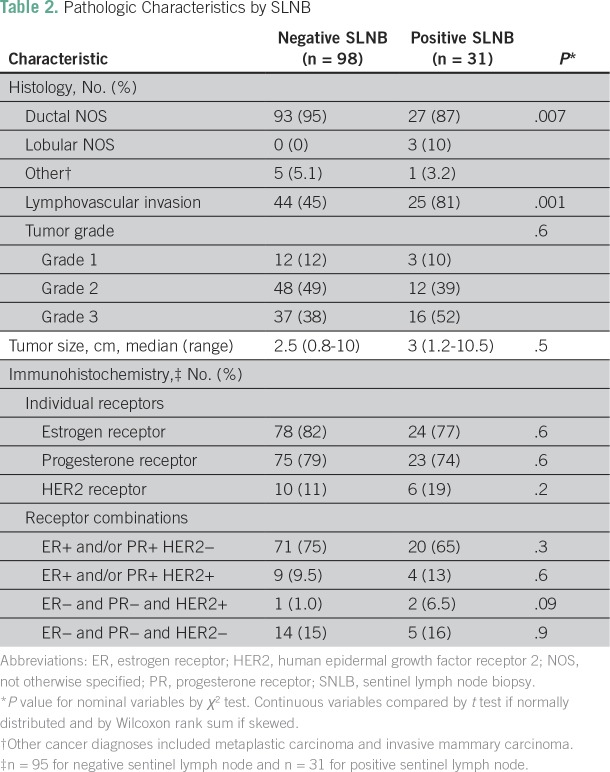
Pathologic Characteristics by SLNB

Of the 129 patients included in our study, 126 underwent IHC for ER, PR, and HER2 receptors. Rates of ER+, PR+, HER2+, and triple-negative disease were 81%, 78%, 13%, and 15%, respectively. A subset analysis among the 65 black patients showed similar results, with ER+, PR+, HER2+, and triple-negative disease rates of 80%, 77%, 17%, and 14%.

Seventy-nine patients were observed for at least 2 years after surgery. Of these, two (3.2%) of the 62 patients with a negative SLNB and two (12%) of the 17 patients with a positive SLNB had a regional recurrence within 2 years, giving an overall 2-year regional recurrence rate of 5.1%. Table 3 lists the clinical characteristics of the four patients who had a regional recurrence. All four of these patients received both adjuvant chemotherapy and radiation therapy for the treatment of their initial breast cancer.

**Table 3:**
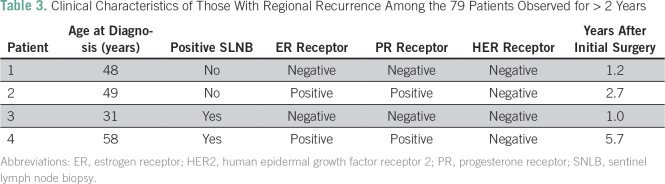
Clinical Characteristics of Those With Regional Recurrence Among the 79 Patients Observed for > 2 Years

## DISCUSSION

The SLNB procedure has been shown to be the standard of care for the management of early-stage breast cancer, with many trials showing that the procedure leads to less morbidity than ALND and results in no difference in overall survival.^14-17^ Despite the widespread adoption of this technique in HICs, there have been few published reports of successful programs from Africa. One cohort of 115 patients from Tunisia who underwent both an SLNB and an ALND reported an SLN identification rate of 97.3% and a false-negative rate of 2.6%.^18^ However, no data have been published from Eastern Africa. To our knowledge, our study, from a single center in Nairobi, Kenya, is the first to report on the pathology and recurrence of patients who underwent an SLNB in this region.

In this study of 129 women with stage I and II breast cancer who underwent an SLNB in a Kenyan hospital between 2008 and 2017, we found that 31 (24%) of the 129 patients had a positive SLNB, including 10 (14%) of the 73 patients with stage I disease and 21 (38%) of the 56 patients with stage II disease. Moreover, one or more additional positive lymph nodes were found in 21 (91%) of the 23 SLNB-positive patients who underwent ALND. The overall SLNB positivity rate was similar to those of cohorts from HICs, which report SLNB positivity rates that range from 24.4% to 28.5%.^15,19^ In addition, we described the 2-year locoregional recurrence rates after treatment with this procedure.

Overall, the regional recurrence rate for patients in our study who were observed for at least 2 years after surgery was 5.1%. This recurrence rate is significantly better than that of the National Surgical Adjuvant Breast and Bowel Project B-04 trial, which randomly assigned women with nonpalpable axillary lymph nodes to radical mastectomy, total mastectomy plus regional radiotherapy, or simple mastectomy alone and showed no survival benefit among the three treatment arms but demonstrated an axillary lymph node recurrence rate of 18.6% among women treated with total mastectomy alone.^20^ Although our results are preliminary, they suggest that the SLNB program at AKUH is successful in identifying women with positive axillary lymph nodes and removing them through ALND compared with treating them with a total mastectomy alone. However, the 2-year recurrence rate is still much higher than 0.7% to 2.4% at 2 years and 5.3% to 6.2% at 10 years, as reported in the Z0011 trial, which assessed the need to complete an ALND among patients with a positive SLNB and found no difference in local recurrence-free survival between patients who underwent an ALND and those who did not. Importantly, however, in the Z0011 trial, 96% of patients were treated with systemic adjuvant therapy and 100% of patients received radiotherapy, compared with our cohort, in which 60% received systemic adjuvant chemotherapy, 85% of eligible women received endocrine therapy, and 62% received radiotherapy.^21^ This discrepancy highlights the need for AKUH and other cancer centers in the region to ensure that patients receive appropriate medical and radiation therapy in addition to cancer surgery.

Interestingly, however, all four patients who had a recurrence received both adjuvant chemotherapy and radiotherapy, suggesting that the increased recurrence rates in the AKUH cohort may be driven partially by the characteristics of the primary tumor. Compared with the Z0011 trial and others from HICs, the cohort at AKUH had more black participants, larger tumors, more stage II disease, and greater rates of lymphovascular invasion,^15,21^ which may have led to increased relapse rates in the AKUH cohort compared with those in HICs. This highlights the need for primary cancer management research in the region to explain the difference in outcomes and to determine if the most appropriate way to manage early-stage disease in Africa is the same as in HICs, or must be modified.

One of the strengths of our study was the completeness of our database, which included IHC for > 97% of participants and had < 3% missing values for any included variable. Another strength was the availability of follow-up data for 2 years after surgery on > 60% of our cohort, which allowed us to document the incidence of early recurrence after SLNB and ALND. 

The limitations of our study include its small sample size, the retrospective study design, and our primary reliance on two highly skilled surgeons, which limits the generalizability of our results to all centers within the region. In addition, we did not systematically collect data on common complications of SLNB and ALND, such as lymphedema and axillary numbness, which prevented us from being able to assess the impact of SLNB on these important causes of morbidity.

In conclusion, to our knowledge, our study describes for the first time the clinical and pathologic characteristics and early recurrence experience of patients with stage I and stage II breast cancer who underwent an SLNB in East Africa. This study shows that although some tumor characteristics are similar between our cohort and cohorts from HICs, early recurrence rates seem to be higher at AKUH. To explain this discrepancy in recurrence rates, more primary data on the treatment of early breast cancer in Africa are needed.
